# Biotic and Abiotic Drivers of Phenotypic Diversity in the Genus *Lupinus* (Fabaceae)

**DOI:** 10.3390/plants15030456

**Published:** 2026-02-02

**Authors:** Mateo Burke Irazoque, Mónica Moraes R., Sissi Lozada-Gobilard

**Affiliations:** 1 Carrera de Biología, Universidad Mayor de San Andrés, La Paz 10077, Bolivia; mburkei2@fcpn.edu.bo; 2 Herbario Nacional de Bolivia, Instituto de Ecología, Universidad Mayor de San Andrés, La Paz 10077, Bolivia; mmoraes@fcpn.edu.bo; 3 Biodiversity Unit, Department of Biology, Lund University, 223 62 Lund, Sweden

**Keywords:** pollination, trait selection, phenotypic integration, reproductive success, local adaptation, lupine

## Abstract

The genus *Lupinus* (Fabaceae) represents an exceptional model system for studying evolutionary processes mediated by pollinators and environmental factors. This review synthesizes evidence on phenotypic variability of floral traits, trait selection by biotic and abiotic factors, and the eco-evolutionary implications of these interactions. Pollinators shape floral traits through specialized selection that optimizes pollination efficiency while maintaining plasticity toward autogamy under pollinator scarcity. At the same time, abiotic pressures drive adaptations to climate and elevation, which *Lupinus* achieves through phenotypic plasticity, such as adjusting flowering time, and through mutualisms with soil microbes that reduce abiotic stress. Ecological implications reveal contrasting dynamics, where native species sustain specialized pollination networks, while invasive species such as *L. polyphyllus* Lindl. disrupt these interactions through competitive exclusion. Thus, these two factors collectively drive *Lupinus* phenotypic diversity through specialized adaptations and plasticity. Finally, we emphasize the need for integrated studies combining genomics and ecological modeling to decipher the adaptive mechanisms of this genus.

## 1. Introduction

The relationship between plants and pollinators is a crucial ecological process for maintaining the structure and function of ecosystems, as well as their diversity [1]. Approximately 90% of flowering plants depend to some degree on animal vectors for their pollination [2]. Consequently, this form of pollination has been a key driver in angiosperm evolution, promoting the diversification of reproductive strategies and morphological adaptations [3]. Thus, in many species, both phenotypic variability and seed production are largely dependent on this relationship, highlighting its role in conserving the course of gene flow and reproductive success [4]. Therefore, the loss of pollinators leads to a reduction in the variability of flowering plants, making pollinator conservation a crucial factor in preserving ecological balance [5].

Among the most significant morphological adaptations linked to pollinator specialization is floral symmetry [6]. Zygomorphic flowers, characterized by their bilateral symmetry, emerged convergently in diverse angiosperm lineages as an adaptation to various climate factors and interactions with specialized pollinators [6,7]. This morphology favors the precise deposition of pollen on specific areas of the pollinator’s body, maximizing transfer efficiency and minimizing its loss during the process [8]. Furthermore, their structural complexity acts as a filter, restricting access to generalist pollinators and favoring those with specialized morphological adaptations and behavioral adaptations [6]. A high degree of floral specialization has been a key driver of diversification, particularly within the legume family (Fabaceae) [9,10]. This trend is exemplified by the subfamily Faboideae, which exhibits a highly conserved zygomorphic floral architecture central to its specialized pollination systems, which is particularly notable in the genus *Lupinus*, where zygomorphic flowers are optimized for specific pollinators [9,10].

However, the evolutionary trajectory of floral traits is not shaped by selective agents alone; it is also channeled and constrained by intrinsic organismal factors [11]. Genetic architecture plays a fundamental role; phenomena such as pleiotropy can create evolutionary trade-offs, limiting the independent evolution of floral and vegetative characters [12]. Similarly, linkage disequilibrium can maintain correlations between traits, like flower color and corolla shape, even if not all are directly favored by pollinators in a given context [13]. Furthermore, developmental and phylogenetic constraints inherited from ancestral legume lineages may bias the morphological variation available for selection, explaining the conserved zygomorphic ground plan in Faboideae despite niche diversification [14]. Phenotypic plasticity may act as a compensatory strategy, enabling rapid adjustment in malleable traits such as flowering time when evolutionary responses in developmentally fixed traits are constrained by genetic or phylogenetic limitations [15,16]. Finally, the evolution of attractive floral signals is inevitably also shaped by antagonistic interactions, such as herbivores or nectar robbers, which can counter-select for conspicuousness or favor resource allocation to defense over advertisement [17,18]. Therefore, a comprehensive understanding of phenotypic diversity in *Lupinus* requires considering both the external drivers (biotic and abiotic) and these internal constraints and physiological or developmental trade-offs that modulate adaptive outcomes.

**Figure 1:**
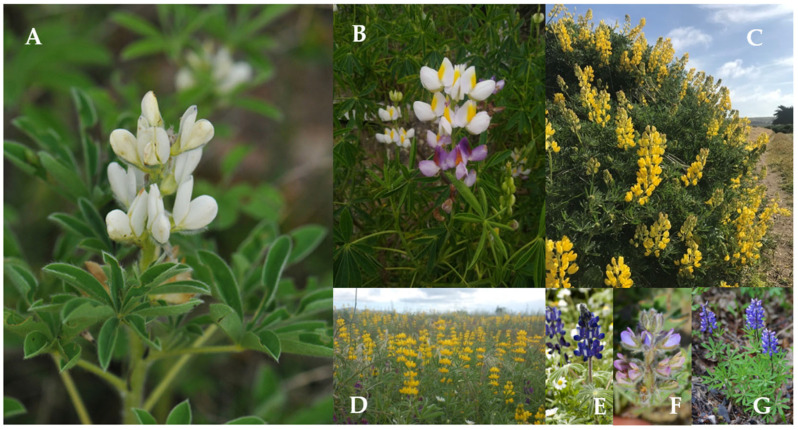
Floral diversity of seven *Lupinus* species discussed in this review. (**A**) *L. albus*. (**B**) *L. mutabilis* Sweet. (**C**) *L. arboreus* Sims. (**D**) *L. luteus* L. (**E**) *L. pilosus* L. (**F**) *L. nipomensis* Eastw. (**G**) *L. arcticus* S. Watson. Image credits: iNaturalist.

*Lupinus*, which comprises more than 200 species concentrated almost entirely in the Americas and the Mediterranean region of Europe, exhibits a wide range of ecological adaptations, and has been introduced and widely naturalized in Europe, Asia, New Zealand, and Australia, some of them considered major weeds such as *L. angustifolius* L., *L. albus* Lindl. and *L. cosentinii* Guss. Refs. [19,20,21] (Figure 1). Pollination in this genus is almost exclusively mediated by insects, particularly among the Andean species [21,22]. These species possess a specialized mechanism, typical of Fabaceae flowers, where the keel only opens when pressure is applied by specific pollinators, thereby ensuring efficient pollen release and transfer [23]. Despite the advantages this mechanism provides for pollinator fidelity and pollen transfer, it also makes them particularly vulnerable to reductions in vector activity due to environmental disturbance [24].

Pollinators may act as selective agents on the floral traits of *Lupinus*, driving variation in flower color, size, and shape [25,26]. Therefore, flowers with a greater number of pollinator-dependent specialized mechanisms are more likely to be found in species visited by a single type of pollinator, whereas pollination regimes with a low or unreliable number of pollinators often favor the evolution of a selfing strategy, which involves its own suite of specialized floral adaptations to ensure autonomous reproduction [22,27].

In addition, vegetative traits, such as plant height and plant architecture, also contribute to the patterns of visitor attraction by enhancing floral visibility or accessibility [22]. *Lupinus* exhibits considerable phenotypic plasticity in these vegetative traits, allowing for rapid and non-genetic adjustments in response to local conditions [28]. Consequently, populations experiencing low pollinator abundance may express different heights or more open architectures, traits that could increase the chance of visitor encounters, highlighting how plasticity mediates the genus’s response to ecological challenges [28,29].

Pollinators not only may drive the floral morphology, but also directly influence plant reproductive success [3,22]. Efficient pollination increases the production of viable seeds, favoring population persistence, while inefficiency decreases fertilization and seedling viability [4,26]. This direct impact of pollinator efficiency on reproductive output can determine changes in the population dynamics and adaptation of plant species [19]. Furthermore, habitat fragmentation and reduction in pollinator diversity can modify ecological and evolutionary processes, affecting the conservation of these species and their ecosystems [30]. In this review, we synthesize available information from academic articles on the influence of pollinators on the phenotypic variation within the genus *Lupinus* (Fabaceae), with emphasis on documented patterns of diversity in floral traits associated with biotic and abiotic factors, the role of these traits in reproductive success, and the ecological and evolutionary implications of these interactions in the context of global change.

## 2. Results

This review synthesizes findings from 63 studies, including 10 that specifically investigate *Lupinus* in the context of phenotypic variability and selection pressures. We categorized the literature based on the primary selective factors examined: biotic (pollinator-mediated), abiotic (environmentally mediated), or a combination of both.

The analysis encompasses 17 *Lupinus* species, representing about 8.5% of the described diversity within a genus that includes more than 200 species [19]. This subset, while limited, includes species of significant ecological and economic importance, from widely cultivated crops (*L. albus* and *L. angustifolius*) to rare endemics (*L. nipomensis*) and aggressive invaders (*L. polyphyllus*).

Biotic factors, primarily pollination mechanisms and interactions, were the focus of 16% of the studies analyzed, covering eight species (Table 1). Research on abiotic factors, such as adaptation to temperature, drought, and elevation, was addressed in 32% of the literature and included 11 species (Table 1). A smaller proportion of studies (6.4%) adopted an integrated approach, examining the interplay between biotic and abiotic selection in *L. argenteus*, *L. luteus*, *L. mutabilis*, *L. perennis* and *L. texensis* (Table 1).

**Figure 2:**
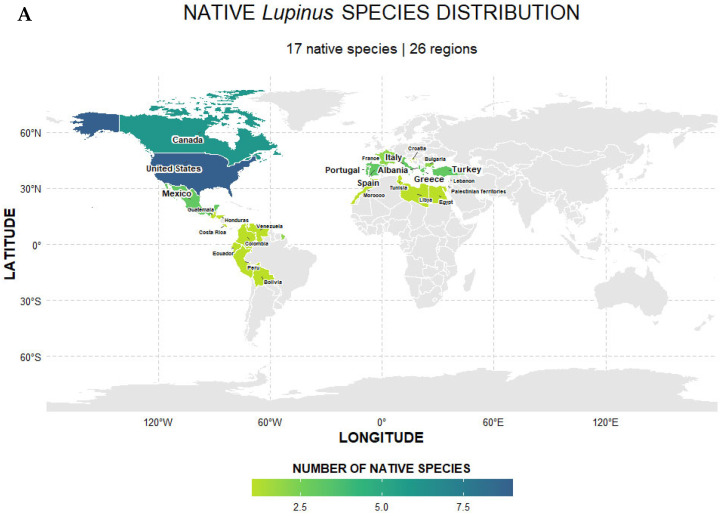

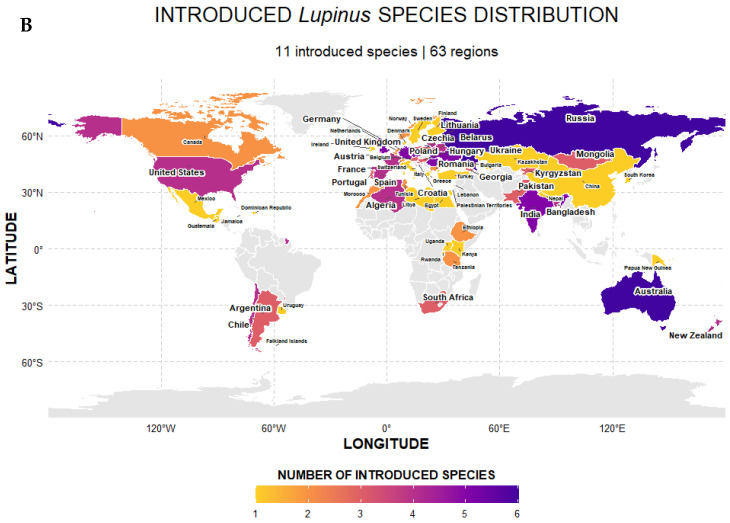
Biogeographic patterns of the 17 studied *Lupinus* species. Maps show species richness in (**A**) native and (**B**) introduced ranges across 89 regions from the POWO database [61]. A total of 349 records were analyzed (144 native, 211 introduced).

The geographic distribution of these 17 species spans native and introduced ranges across the Americas, the Mediterranean, and other regions, as mapped in Figure 2. This biogeographic context is crucial for interpreting the localized adaptation and ecological impacts discussed throughout this review. The following sections detail the documented phenotypic responses to these selective pressures.

### 2.1. Selection of Floral Traits in Lupinus Mediated by Pollinators

*Lupinus* exhibits remarkable phenotypic diversity in floral and vegetative traits, underpinned by a complex genetic control and pollinator interactions [43,60]. Studies on *Lupinus mutabilis* and *L. albus* reveal that variations in flower color, seed size, and phenology arise from processes of hybridization and introgression, along with the presence of rare alleles with adaptive potential [40,57] (Table 1, Figure 1). In addition to these genomic sources of variation, floral and vegetative traits in *Lupinus* exhibit considerable phenotypic plasticity [28,33]. This plasticity allows for flexible responses to selective pressures, but its expression depends on interactions between pollinators and environmental conditions [25,56]. For example, this leads to the selection of yellow corollas that maximize attraction by indicating a greater reward in *Lupinus argenteus*, or the shortening of the flowering duration by its visitors, as observed in *L. albus*, although the latter is capable of self-pollination [32,35].

Pollinators act as key selective agents in the evolution of floral traits in *Lupinus*, favoring visual, tactile, and chemical signals that maximize attraction and reproductive efficiency (Table 1) [28,36]. Studies on *L. argenteus* demonstrate that post-pollination color change directs pollinators towards flowers with active rewards, optimizing their foraging effort [35]. Furthermore, structures such as the floral keel and inflorescence architecture can attract visitors and filter out less effective ones, promoting specialization [28,62,63]. These traits reflect a balance between attraction and efficiency but also expose limitations in the face of environmental disturbances [64].

Floral chemistry emerges as another axis of pollinator-mediated selection [36]. Quinolizidine alkaloids in the pollen of *L. argenteus* and *L. sulphureus* deter pollen thieves while being tolerated by legitimate pollinators, suggesting chemical co-evolution in this group [46]. Evidence suggests that these secondary metabolites function beyond defense, also filtering interactions to optimize pollen flow [36]. In addition, the absence of nectar in many *Lupinus* species implies that pollen acts as the sole reward, a trait that may restrict the attraction of generalist pollinators in degraded environments [31].

Phenology and olfactory signals are also two factors that play a critical role [28,65]. In *L. arcticus*, floral scent regulates flower longevity, ensuring pollination in cold climates, while pollen alkaloids reinforce the fidelity of specialized visitors [28,36]. These adaptations are crucial in habitats with narrow pollination windows, and their effectiveness depends on synchrony with pollinator activity cycles [46]. In situations with few visitors, mixed strategies, such as the synergy of deceptive and honest signals, demonstrate how selection favors complex mechanisms to maximize cross-pollination [25].

Organic farming practices that promote a greater diversity of pollinators can lead to broader phenotypic variability in *L. mutabilis*, thereby buffering populations against the risks associated with overspecialization [64]. Indeed, pollination specialization entails risks, as seen with the keel mechanism in Andean species, which, although efficient, makes them vulnerable to the loss of specific pollinators [24]. Thus, habitat fragmentation can reduce pollinator visits, favoring self-pollination and decreasing genetic diversity [4]. Studies on *L. mutabilis* show that cross-pollination rates can exceed 50% in conserved habitats, but, in altered environments, phenotypic plasticity may not compensate for the lack of pollinators [22]. This dependence underscores the fragility of plant-pollinator interactions in the face of environmental disturbances.

As previously mentioned, the dependence on specialized pollinators has genetic implications for this group [66]. For instance, *L. nanus* exhibits facultative self-pollination, which does not compensate for the loss of genetic diversity associated with pollinator reduction, whereas in *L. arboreus*, outcrossed plants produce 47% more viable seeds than self-pollinated ones, demonstrating that, although some *Lupinus* species have reproductive assurance mechanisms, allogamy remains essential for maintaining adaptive variability [27,34,39]. Pollinator preference for certain phenotypes reinforces this dynamic, but it also reveals a conflict: highly specialized traits can become counterproductive if the preferred pollinators disappear [25].

### 2.2. Selection of Floral Traits in Lupinus Mediated by Abiotic Factors

Abiotic factors interact with biotic pressures, generating local adaptations [22,32]. Two examples of this occur in *L. angustifolius* and *L. mutabilis*; the former accelerates flowering and produces heavier seeds under water stress, while the latter adjusts its phenology to synchronize with pollinators in Andean environments [33,65]. Another response reflecting evolutionary trade-offs occurs in warm environments, where conditions that favor the early flowering of *L. argenteus* reduce the window for interaction with pollinators [35].

Abiotic factors also exert fundamental selective pressure on the reproductive cycles of *Lupinus* (Table 1) [33,41,49]. Studies on *L. albus* demonstrate that high temperatures and long photoperiods accelerate its flowering, while cold conditions inhibit it [42]. Similarly, elevated temperatures can disrupt vernalization requirements in *L. hispanicus*, leading to phenological desynchronization and reduced frost resistance [48]. This phenological plasticity represents a key adaptation to avoid adverse climatic events such as late frosts; however, vernalization limits the distribution of *Lupinus* in regions with mild winters, revealing a significant adaptive trade-off [41].

*Lupinus* also exhibits vegetative adaptations to cope with thermal stress [41]. There are reports that *L. polyphyllus* can tolerate temperatures as low as −5 °C through physiological adjustments, and Andean accessions of *L. mutabilis* display genetic variability for frost resistance (−2 °C to −10 °C) [50,53]. In contrast, *L. luteus* regulates phytohormones, such as ethylene, to combat heat [49]. This diversity of mechanisms may help to explain the broad ecological distribution of the genus and its distinct selective pressures [57].

Similarly, elevation acts as a powerful environmental filter for *Lupinus*; for example, in the Andes, *L. mutabilis* produces seeds resistant to extreme conditions at higher elevations, adapted to increased UV radiation and reduced competition [57]. In the Mediterranean, high elevations select for smaller leaves and earlier flowering in *L. angustifolius* [33]. However, these adaptations can come with costs; for example, in *L. mutabilis*, reproductive success is lower at higher elevations, a pattern associated with reduced pollinator visitation rates and efficiency [22].

Additionally, water availability shapes key traits in *Lupinus* [15]. In *L. albus*, earlier floral development has been observed for greater water efficiency under drought conditions [39]. Following the same trend, *L. angustifolius* illustrates various trade-offs, producing smaller seeds under water stress, affecting seedling vigor [29]. Similarly, soil resource availability and cultivation medium significantly influence root architecture and phenotypic plasticity in *L. angustifolius*, highlighting how edaphic factors drive belowground adaptations [45]. Likewise, in response to abiotic stresses such as nutrient-poor or dry soils, phenotypic plasticity facilitates key adaptations: the recruitment of beneficial rhizospheric microbiota in *L. nipomensis* and the hormonal regulation of growth in *L. polyphyllus* [49,52]. These plastic responses enhance the colonization of diverse and stressful habitats by these two species.

These patterns of floral trait selection in *Lupinus* lead to reproductive plasticity reflecting adaptations to abiotic factors [33]. One study identified a correlation where *L. polyphyllus* in extreme environments produces larger seeds which likely improves seedling establishment at the cost of a lower total seed number, representing a potential evolutionary trade-off [55]. In parallel, *L. argenteus* present phenotypic plasticity in nectar production, eliminating this reward in arid areas to conserve resources, relying solely on pollen as a reward [46]. However, these tactics were less effective at high elevations with scarce pollinators, evidencing how abiotic factors modulate biotic interactions [22].

## 3. Discussion

### 3.1. Drivers of Phenotypic Diversity

This review synthesizes published information on the influence of pollinators on the phenotypic floral variability of *Lupinus*, focusing on trait diversity linked to biotic and abiotic factors, their role in reproductive success, and the eco-evolutionary implications of these interactions. Considering this evidence, the selection of floral traits in *Lupinus* mediated by pollinators reveals a delicate balance between specialization and vulnerability. Adaptations, such as specialized floral keels, defensive alkaloid profiles, and color-change signals, optimize pollination but also generate a critical dependence on specific pollinators, and when they decline, there is a decrease in genetic diversity and reproductive success [27,34]. Simultaneously, abiotic factors, such as temperature, water availability, and elevation, impose strong selective pressures on these traits, often modulating the expression and effectiveness of pollinator adaptations [33,41]. These complex plant–pollinator–environment interactions face new challenges under global change, as phenological desynchronization and the decline of pollinators resistant to metabolites could limit the efficacy of evolved traits [36]. The extensive documented plasticity in *Lupinus* offers hope for resilience, as it can maintain phenotypic diversity and stabilize pollination networks under environmental change. Furthermore, integrating this eco-evolutionary understanding with sustainable agricultural and conservation practices will be key to ensuring the long-term resilience of wild populations and the ecosystems they inhabit [64].

Climate change can decouple the synchrony between floral phenology and pollinator activity, reducing plant reproductive success [33]. Although rare alleles and phenotypic plasticity offer a degree of resilience, rates of climate change may surpass the adaptive capacity of some species [56]. This vulnerability is contingent on the resilience of the pollinator partners themselves. While co-evolved pollinators may possess inherent resistance to historical environmental fluctuations, the unprecedented rate and magnitude of contemporary global changes threaten to surpass such adaptive buffers, potentially leading to the breakdown of even long-standing specialized mutualisms [22,47]. For instance, in *L. albus*, the conservation of specific alleles is crucial for responding to water stress, but allelic persistence depends on connectivity among populations and the maintenance of pollination networks [40].

### 3.2. Evolutionary Mechanisms and Plasticity

As previously mentioned, this synthesis confirms that pollinators and abiotic factors are fundamental drivers shaping phenotypic diversity in *Lupinus*; however, a comprehensive understanding of these patterns requires integrating the evolutionary mechanisms that facilitate or constrain adaptive changes. The documented trait associations likely arise not only from direct selection by these external agents but also from the organism’s intrinsic genetic architecture [11]. Genetic correlations, mediated by linkage disequilibrium or pleiotropy, can couple floral traits with physiological adaptations, such as drought tolerance, potentially limiting their independent evolution [12,67]. For example, the consistent correlation between specific floral pigments and quinolizidine alkaloid profiles across species suggests a genetic linkage that packages chemical defense with visual advertisement [11,46]. This intrinsic constraint likely limits evolutionary pathways and helps explain why shifts toward generalist pollination systems are rare in this genus, even when pollinator populations decline.

At the same time, the evolution of attractive and efficient floral structures is inherently balanced against pressures from antagonists, such as herbivores and pollen thieves, which exert counter-selection on resource allocation and trait expression [68,69]. Therefore, the optimal floral phenotype in any population represents a compromise between mutualistic and antagonistic selection, all operating within the bounds of developmental and phylogenetic constraints inherited from the legume lineage [16,17]. In this light, the well-documented phenotypic plasticity in traits like flowering time or growth form emerges not merely as a direct adaptation, but as a crucial compensatory mechanism enabling rapid adjustment when deep-rooted genetic or phylogenetic inertia limits evolutionary change in more constrained structural traits [16]. To move from pattern to process, future research should employ genomic tools—such as QTL mapping and GWAS in key species—to directly disentangle pleiotropic effects from linkage disequilibrium [13,70]. Furthermore, comparative phylogenomic approaches and manipulative field experiments that simultaneously vary pollinator access and antagonist pressure are needed to quantify the net selective balance and reveal the full spectrum of evolutionary forces acting on *Lupinus* phenotypes [68].

### 3.3. Knowledge Gaps and Research Bias

This ecological and evolutionary complexity is greatly biased by the available knowledge for the genus *Lupinus*. Despite its broad global distribution and great diversity of over 200 species, pertinent studies have concentrated on a small subset of taxa, as illustrated by the 17 species included in this study (Table S1 and Figure 2) [19,20]. This selection reflects a bias towards species with immediate economic relevance (e.g., *L. albus* for food, *L. angustifolius* for forage, Table S1) or those inhabiting regions with greater research funding. Consequently, a vast information gap exists regarding the reproductive biology, pollinator interactions, and phenotypic plasticity of the majority of *Lupinus* species, many of which could harbor key adaptations and critical vulnerabilities yet to be discovered. Increasing knowledge on additional species is crucial for understanding the true breadth of this group’s responses to global change and for designing effective conservation strategies beyond a few species of current interest.

### 3.4. Ecological and Evolutionary Implications

In parallel to biotic factors, abiotic selection in *Lupinus* reveals a complex balance between local specialization and phenotypic plasticity. Adaptations such as frost tolerance or water efficiency are advantageous under stable conditions but could become limiting in the face of abrupt climatic changes [39,53]. Likewise, the desynchronization between floral phenology and pollinator activity at high elevations is concerning [22,33]. Future research should harness emerging genomic resources, such as the relatively compact and tractable reference genome of economically significant species like *L. mutabilis*, to develop targeted genetic tools [71]. Integrating these genomic studies with eco-evolutionary models will be key to predicting adaptive responses, prioritizing both the conservation of pollinators and intraspecific genetic diversity [15]. This approach will facilitate more specific studies on adaptation, selection, and the identification of quantitative trait loci (QTLs) underlying key agronomic and ecological traits, as demonstrated for drought tolerance and alkaloid content [40,41,46]. Similarly, managing trade-offs, such as seed size versus seed number, will be crucial for maintaining both ecosystem services and agricultural productivity under changing climatic scenarios [55].

The balance between conservation and control is critical for *Lupinus* [51]. Rare species require habitat restoration and the protection of specialized pollinators, while invasive species demand active management to mitigate their impacts [37,72]. Furthermore, understanding the genetic basis of the adaptation of *Lupinus* will be essential for predicting responses to climate change [73]. This dual approach—conserving rare and controlling invasive species—must guide management strategies in future scenarios.

*Lupinus* species can profoundly alter the ecosystems where they naturalize [65]. This is exemplified by the alteration of native plant communities in Europe by *L. polyphyllus*, which, by tolerating poor soils and attracting generalist pollinators, can competitively exclude native co-flowering species [53,54]. However, impacts vary depending on the context; *L. arboreus* acts as a key resource for bees by offering abundant pollen, an adaptation to saline and infertile soils [34]. These cases illustrate how the genus can act as an ecosystem engineer in diverse ways.

Associations with soil microorganisms are key for the adaptation of *Lupinus* to stressful environments [44]. These symbioses enhance tolerance to drought and nutrient-poor soils, but their efficacy varies according to local conditions [44,52]. In degraded habitats, these mutualistic interactions become more relevant, allowing species such as *L. nipomensis* to survive under climatic stress [51]. However, dependence on specific microbiota could limit their expansion into areas with altered soils, revealing a trade-off between plasticity and specialization [57].

The floral traits of *Lupinus* also reflect intense adaptation and specialization for its pollinators [38,64]. Variation in characteristics such as floral tube length promotes reproductive isolation, driving adaptive radiations where different species attract specific groups of pollinators [36,74]. However, this high degree of specialization creates a latent vulnerability, as evidenced by *L. nipomensis*, where the decline of its specific pollinator risks collapsing the reproductive isolation mechanism that their interaction originally reinforced [51]. Thus, while this high degree of reciprocal specialization has fostered diversification, it also creates risk from anthropogenic disturbances [36,46].

Cross-pollination in *Lupinus* maintains high genetic diversity, facilitating rapid adaptations [27]. In *L. perennis*, both population density and abiotic conditions such as weather modulate pollinator behavior, directly affecting pollination efficiency and genetic outcomes [58]. Consequently, genetic diversity is influenced by both pollinator effectiveness and specificity, such that populations serviced by effective, cross-pollinating specialists can maintain high genetic variation, while those visited by a greater variety of generalist pollinators may experience increased heterospecific pollen deposition, which can impair reproductive success despite high pollinator visits [47,75]. Nevertheless, the role of plasticity in ensuring resilience has clear boundaries, leading to divergent outcomes. High plasticity can itself drive significant impact, as seen in invasive species like *L. polyphyllus*, which reduce native biodiversity [72]. Conversely, when plasticity is limited or ineffective, specialized species like *L. nipomensis* face a disproportionately high risk of extinction [51]. These contrasts highlight that the genus’s resilience depends both on its flexibility and on the conservation of its biotic interactions [51,53]. Moreover, *L. polyphyllus* is not an isolated case; other congeners like *L. angustifolius*, *L. albus*, *L. luteus* and *L. pilosus* have become significant weeds in continental areas such as Australia, underscoring a recurring management challenge for the genus (Table S1) [76].

### 3.5. Methodological Considerations and Future Directions

A key consideration when interpreting these findings is the methodological diversity among the reviewed studies. The term “pollinator” often encompasses the entire floral visitor assemblage observed, as many studies use visitation rates as a proxy for pollination effectiveness. This approach can blur the distinction between effective pollinators and mere visitors, potentially overestimating the selective role of some agents [25,38]. Future studies quantifying pollen deposition or plant reproductive success directly in relation to visitor identity and behavior are needed to refine our understanding of true pollinator-mediated selection.

The ecological and evolutionary implications of *Lupinus* reveal a complex system where co-evolution, plasticity, and vulnerabilities are intertwined [22,36]. While its capacity to modify ecosystems and adapt to environmental changes is remarkable, its dependence on specific interactions poses critical challenges [47,53]. Future research should prioritize monitoring plant–pollinator networks in altered habitats, identifying resilience thresholds in specialist species, and integrating genomic tools with environmental and ecological modeling to identify adaptive potential, predict ecological trajectories, and assess invasiveness. The effective conservation of the genus will require integrating these aspects, recognizing its dual role as a keystone species and a potential invader, and its value as a model for understanding eco-evolutionary responses to global change [51,53]

## 4. Materials and Methods

A systematic literature search was conducted to identify relevant studies on the genus *Lupinus*, focusing on floral traits, abiotic and biotic factors, and pollination. The search was performed in May 2025 using two primary academic databases: Google Scholar and Web of Science (Core Collection).

The following key search terms were employed: “Lupinus”, “Phenotypic Variability”, “Phenotypic Variation”, “pollinator”, “pollinators influence”, “abiotic factors”, “biotic factors”, and “pollination”. These terms were used individually and in combination to maximize the retrieval of pertinent literature. The search was not restricted by publication year to ensure a comprehensive historical coverage of the topic.

The initial search results were screened based on their titles and abstracts. Studies were selected for full-text review if they provided specific empirical data or substantial reviews concerning the interplay between phenotypic variability (including floral morphology, color, scent, and reproductive strategies) and environmental or pollinator-mediated selection pressures in *Lupinus* species. The studies were screened to include those that either directly measured selection on floral traits or provided indirect evidence of selection through experiments, trait–fitness correlations, or ecological trait variation. This selection process culminated in the body of literature synthesized in this review.

To complement the literature data, the global distribution of the *Lupinus* species identified in the selected studies was mapped. Geographic visualization (Figure 2) was performed using R software (version 4.3.2; Posit, Boston, MA, USA) with the following packages: ggplot2, sf, rnaturalearth, ggrepel, countrycode, viridis, and patchwork. Geographic occurrence data were retrieved from the Plants of the World Online (POWO) database [61]. The distribution records for each species were extracted and consolidated to visualize their native and introduced ranges, providing a biogeographic context for the ecological and evolutionary patterns discussed in this review. Additionally, a summary of pollination systems for the studied species is provided in Supplementary Materials (Tables S1 and S2).

## 5. Conclusions

As this review reveals, pollinators exert essential selective pressure on the floral traits of *Lupinus*, ranging from floral morphology to reproductive strategies. However, phenotypic variability is not only shaped by an interplay of biotic factors (e.g., pollinator preferences) but also by abiotic factors (e.g., elevation and climate), soil microbiota interactions and inherent plasticity, which contribute to the genus’s resilience. However, this adaptive capacity is challenged ecologically by invasive species like *L. polyphyllus*, which disrupt native pollination networks. At the same time, significant knowledge gaps hinder researchers’ ability to predict and manage these dynamics.

Critical gaps persist, particularly in understanding the genetic basis of trait–pollinator linkages in understudied Andean species, the limits of plasticity under climate extremes, and the long-term impact of invasions on pollinator functional diversity. Furthermore, key biotic dynamics—such as interactions with the rhizosphere microbiome and with co-flowering plants—remain underexplored despite their potential to modulate adaptation.

To address these gaps, future research must integrate genomic tools with field experiments to decipher the molecular basis of adaptation and plasticity. Priorities include investigating climate-driven phenological shifts in high-mountain species like *L. mutabilis*, establishing long-term monitoring to quantify invasion impacts, and developing conservation strategies that explicitly restore biotic interactions and incorporate genomic insights into the management of both rare and invasive species. Finally, future studies should aim to disentangle the net selection balance imposed by pollinators versus antagonists, and to quantify the role of genetic constraints in shaping evolutionary responses.

## Figures and Tables

**Table 1:** Documented biotic, abiotic, and combined selective pressures on floral traits in *Lupinus* species.

**Biotic Factors**		
**Species**	**Trait**	**Reference**
*L. albus* L.	Pollen is the only resource, benefitting specialist pollinators	[31]
	Shortening of flowering duration due to visitor activity	[32]
*L. angustifolius* L.	Pollen is the only resource which benefits specialist pollinators	[31]
	Synchronization between floral phenology and pollinator activity	[33]
*L. arboreus* Sims.	Cross-pollination increases seed viability	[34]
*L. argenteus* Pursh	Yellow color maximizes flower attraction	[35]
	Produces metabolites that filter pollinators	[36]
*L. nanus* Douglas ex Benth.	Self-pollination increases when pollinator numbers decline	[27]
*L. perennis* L.	Distribution depends largely on its pollinator	[37]
*L. pilosus* L.	Presence of deceptive and honest signals under low pollinator numbers	[25]
*L. sp.*	Shape and size of the corolla affect the number of visitors	[38]
**Abiotic Factors**		
**Species**	**Trait**	**Reference**
*L. albus* L.	High drought tolerance	[39]
	Possesses private alleles for water stress	[40]
	Vernalization is an adaptation to cold	[41]
	Flowering time is altered by temperature and light exposure	[42]
	Environmental factors increase genetic and phenotypic variability	[43]
*L. angustifolius* L.	Production of smaller seeds under drought	[29]
	Regulation of microbiota enables resistance to poor soils and droughts	[44]
	Environmental factors increase genetic and phenotypic variability	[43]
	Soil resource availability (nutrients, water, texture) and cultivation medium significantly influence root architecture (length, branching, biomass) and phenotypic plasticity	[45]
*L. arcticus* S. Watson	Scent production regulates pollination in cold climates	[28]
*L. bakeri* Greene	Alkaloids in pollen deter pollen thieves	[46]
*L. elegans* Kunth	Climatic factors regulate its phenotypic variability	[47]
*L. hispanicus* Boiss. & Reut.	Altered phenology and vernalization response under elevated temperatures, potentially leading to flowering desynchronization and reduced frost resistance	[48]
*L. luteus* L.	Hormonal regulation to withstand climatic factors	[49]
*L. mutabilis* Sweet	Elevation affects reproductive success and inflorescence morphology	[22]
	Frost resistance down to −10 °C	[50]
*L. nipomensis* Eastw	Mutualistic interaction enabling growth under climatic stress	[51]
	Adjustment of rhizospheric microbiota in extreme habitats	[52]
*L. polyphyllus* Lindl.	Tolerance of temperatures down to −5 °C	[53]
	Physiological adjustment to tolerate poor soils	[54]
	Hormonal regulation to withstand climatic factors	[49]
	Increase in seed size in extreme environments	[55]
*L. sulphureus* Douglas	Alkaloids in pollen deter pollen thieves	[46]
**Combined Factors**		
**Species**	**Trait**	**Reference**
*L. argenteus* Pursh	Alkaloids in pollen deter pollen thieves; nectar production ceases in arid sites	[46]
*L. luteus* L.	Flower plasticity is a response to pollinator preferences and climatic factors	[56]
*L. mutabilis* Sweet	Color and shape are traits that co-evolve with pollinators; Seed adaptation to higher radiation and lower competition at higher elevations	[57]
*L. perennis* L.	Pollinator behavior, especially in small/dense populations, and abiotic conditions, such as weather, light, temperature, interact to determine pollination efficiency, seed set, and genetic diversity.	[58]
*L. texensis* Hook.	Pollinator-mediated outcrossing and soil resources (nutrients, water, light) interact to influence early-life survival, reproductive success, and inbreeding depression across populations.	[59]

## Data Availability

No new data were created for this study.

## Supplementary Materials

The following supporting information can be downloaded at: https://www.mdpi.com/article/10.3390/plants15030456/s1, Table S1: Documented Uses and Applications of the 17 *Lupinus* Species Reviewed in this Study, Table S2: Pollination Systems in *Lupinus* Species.
