# Supplementary material for: Biotic and Abiotic Drivers of Phenotypic Diversity in the Genus Lupinus (Fabaceae)
Source: Plants (Basel). 2026 Feb 2;15(3):456. doi: 10.3390/plants15030456 (PMC12899042; doi:10.3390/plants15030456)
Supplement: Supplementary file 1 [file plants-15-00456-s001.zip › plants-4080052-supplementary.pdf]

Supplementary Table S1. Documented Uses and Applications of the 17 *Lupinus* Species Reviewed in this Study.

Species	Primary Use/s	Reference
L. albus	Human consumption (high protein), forage, green manure and Australian weeds	[1,2]
L. angustifolius	Human nutrition, animal feed (Australian feedstock), rotation crop and Australian weeds	[1,3,4]
L. arboreus	Ecological (highly invasive species), soil amendment, and stabilization (fast growth, high N-fixing capacity)	[5]
L. arcticus	Research (seed germination studies of 10,000-year-old seeds) and ecological role (insects and squirrels depend on it)	[6]
L. argenteus	Forage and ecological restoration (native to North American prairies; N-fixation in poor and high-elevation soils)	[7]
L. bakeri	Ecological (limited information available; native to North America)	[8]
L. elegans	Ornamental (showy colors) and ecological restoration	[9,10]
L. hispanicus	Forage potential assessment (green mass/grain) under Mediterranean winter conditions and food (high protein content)	[11,12]
L. luteus	Human consumption, animal feed (primarily seeds), N-fixation and Australian weeds	[1,13]
L. mutabilis	Food (high nutritional value) and cold resistance	[14,15]
L. nanus	Ornamental (rock gardens) and cover crop	[16]
L. nipomensis	Conservation (endangered/endemic to California)	[17]
L. perennis	Ecological (host plant for three endangered butterfly species)	[18]
L. pilosus	Agronomic potential	[19]
L. polyphyllus	Ornamental and invasive species	[1,20]
L. sulphureus	Ecological restoration (tolerance to dry and poor soils) and agronomic potential	[21]
L. texensis	Source of antioxidant flavonoids and nitrogen-fixing cover crop	[22,23]

Supplementary Table S2. Pollination Systems in Lupinus Species.

Species	Pollination System	Additional Information	Reference
L. albus	Primarily autogamous / Tripping with pollinators	Mostly pollinated by bees. Self-fertile. Bees exert sufficient pressure to activate flowers and release pollen	[24]
L. angustifolius	Tripping / Capable of self-pollination	Mostly pollinated by bees	[24]
L. arboreus	Tripping	Mostly pollinated by bees. Slightly autogamous	[5,25]
L. argenteus	Tripping	Mostly pollinated by bumblebees	[26]
L. luteus	Tripping / Autogamous	Mostly pollinated by bees. Strong self-fertility trait	[24]
L. mutabilis	Tripping	Mostly by bees	[24]
L. nanus	Tripping / Autogamous	Capable of self-pollination, but limited by bee pollination	[16]
L. pilosus	Tripping	Primarily self-pollinated, with limited bee pollination	[27]

References

1. Australian Government The Biology of Lupinus L. 2013.
2. Huyghe, C. White Lupin (*Lupinus albus* L.). *Field Crops Res.* **1997**, *53*, 147–160, doi:10.1016/S0378-4290(97)00028-2.
3. Lemus-Conejo, A.; Rivero-Pino, F.; Montserrat-de La Paz, S.; Millan-Linares, M.C. Nutritional Composition and Biological Activity of Narrow-Leafed Lupins (*Lupinus angustifolius* L.) Hydrolysates and Seeds. *Food Chem.* **2023**, *420*, 136104, doi:10.1016/j.foodchem.2023.136104.
4. Heenan, D.P.; Taylor, A.C.; Chan, K.Y.; McGhie, W.J.; Collins, D.; Lill, W.J. The Impact of Long-Term Rotation, Tillage and Stubble Management on Lupin (*Lupinus angustifolius*) Productivity. *Field Crops Res.* **2000**, *67*, 11–23, doi:10.1016/S0378-4290(00)00078-2.
5. Stout, J.C.; Kells, A.R.; Goulson, D. Pollination of the Invasive Exotic Shrub *Lupinus arboreus* (Fabaceae) by Introduced Bees in Tasmania. *Biol. Conserv.* **2002**, *106*, 425–434, doi:10.1016/S0006-3207(02)00046-0.
6. Porsild, A.E.; Harington, C.R.; Mulligan, G.A. *Lupinus arcticus* Wats. Grown from Seeds of Pleistocene Age. *Science* **1967**, *158*, 113–114, doi:10.1126/science.158.3797.113.
7. Goergen, E.; Chambers, J.C.; Blank, R. Effects of Water and Nitrogen Availability on Nitrogen Contribution by the Legume, *Lupinus argenteus* Pursh. *Appl. Soil Ecol.* **2009**, *42*, 200–208, doi:10.1016/j.apsoil.2009.04.001.
8. Heiling, J.M.; Cook, D.; Lee, S.T.; Irwin, R.E. Pollen and Vegetative Secondary Chemistry of Three Pollen-rewarding Lupines. *Am. J. Bot.* **2019**, *106*, 643–655, doi:10.1002/ajb2.1283.
9. Didur, I.M.; Prokopchuk, V.M.; Pantsyreva, H.V. Investigation of Biomorphological and Decorative Characteristics of Ornamental Species of the Genus *Lupinus L. Ukr. J. Ecol.* **2019**, *9*, 287–290, doi:10.15421/2019_92.
10. Lara-Cabrera, S.I.; Alejandre-Melena, N.; Medina-Sánchez, E.I.; Lindig-Cisneros, R. Genetic Diversity in Populations of *Lupinus elegans* Kunth, Implications for Ecological Restoration. *Rev. Fitotec. Mex.* **2009**, *32*, 79–86, doi:10.35196/rfm.2009.2.79-86.

11. Lema, M.; Soengas, P. Delve into the Potential of Wild Populations of *Lupinus hispanicus* **2022**. Preprint. https://doi.org/10.21203/rs.3.rs-1789281/v1
12. Bielski, W.; Surma, A.; Belter, J.; Kozak, B.; Książkiewicz, M.; Rychel-Bielska, S. Molecular Dissection of the Genetic Architecture of Phenology Underlying *Lupinus hispanicus* Early Flowering and Adaptation to Winter- or Spring Sowing. *Sci. Rep.* **2025**, *15*, 15324, doi:10.1038/s41598-025-00096-1.
13. Musco, N.; Cutrignelli, M.I.; Calabrò, S.; Tudisco, R.; Infascelli, F.; Grazioli, R.; Lo Presti, V.; Gresta, F.; Chiofalo, B. Comparison of Nutritional and Antinutritional Traits among Different Species (*Lupinus albus* L., *Lupinus luteus* L., *Lupinus angustifolius* L.) and Varieties of Lupin Seeds. *J. Anim. Physiol. Anim. Nutr.* **2017**, *101*, 1227–1241, doi:10.1111/jpn.12643.
14. Barda, M.S.; Chatzigeorgiou, T.; Papadopoulos, G.K.; Bebeli, P.J. Agro-Morphological Evaluation of Lupinus Mutabilis in Two Locations in Greece and Association with Insect Pollinators. *Agriculture* **2021**, *11*, 236, doi:10.3390/agriculture11030236.
15. Chalampuente-Flores, D.; Mosquera-Losada, M.R.; Ron, A.M.D.; Tapia Bastidas, C.; Sørensen, M. Morphological and Ecogeographical Diversity of the Andean Lupine (*Lupinus mutabili*s Sweet) in the High Andean Region of Ecuador. *Agronomy* **2023**, *13*, 2064, doi:10.3390/agronomy13082064.
16. Karoly, K. Pollinator Limitation in the Facultatively Autogamous Annual, *Lupinus nanus* (Leguminosae). *Am. J. Bot.* **1992**, *79*, 49–56, doi:10.1002/j.1537-2197.1992.tb12622.x.
17. Nguyen, P.T.; Luong, J.C.; Wishingrad, V.; Stratton, L.; Loik, M.E.; Meyer, R.S. Soil Biome Variation of *Lupinus nipomensis* in Wet-cool vs. Dry-warm Microhabitats and Greenhouse. *Am. J. Bot.* **2025**, *112*, e70020, doi:10.1002/ajb2.70020.
18. Framer, E. The Short-Term Effect of Fire on *Lupinus perennis* (L.). *Nat. Areas J.* **1996**, *16*, 41–18.
19. Shelef, O.; Ben-Simchon, E.; Sternberg, M.; Cohen, O. Agronomic Estimation of Lupin (*Lupinus pilosus* L.) as a Prospective Crop. *Agronomy* **2024**, *14*, 2804, doi:10.3390/agronomy14122804.
20. Prass, M.; Ramula, S.; Jauni, M.; Setälä, H.; Kotze, D.J. The Invasive Herb *Lupinus polyphyllus* Can Reduce Plant Species Richness Independently of Local Invasion Age. *Biol. Invasions* **2022**, *24*, 425–436, doi:10.1007/s10530-021-02652-y.
21. Cook, D.; Lee, S.T.; Gardner, D.R.; Pfister, J.A.; Welch, K.D.; Green, B.T.; Davis, T.Z.; Panter, K.E. The Alkaloid Profiles of *Lupinus sulphureus*. *J. Agric. Food Chem.* **2009**, *57*, 1646–1653, doi:10.1021/jf803468q.
22. Andam, C.P.; Parker, M.A. Novel Alphaproteobacterial Root Nodule Symbiont Associated with *Lupinus texensis*. *Appl. Environ. Microbiol.* **2007**, *73*, 5687–5691, doi:10.1128/AEM.01413-07.
23. Zhang, Z.; Yuan, W.; Wang, P.; Grant, G.; Li, S. Flavonoids from *Lupinus texensis* and Their Free Radical Scavenging Activity. *Nat. Prod. Res.* **2011**, *25*, 1641–1649, doi:10.1080/14786419.2010.523423.
24. Williams, I.H. The Pollination of Lupins. *Bee World* **1987**, *68*, 10–16, doi:10.1080/0005772X.1987.11098904.
25. Kittelson, P.M.; Maron, J.L. Outcrossing Rate and Inbreeding Depression in the Perennial Yellow Bush Lupine, *Lupinus arboreus* (Fabaceae). *Am. J. Bot.* **2000**, *87*, 652–660, doi:10.2307/2656851.
26. Heiling, J.M.; Cook, D.; Lee, S.T.; Irwin, R.E. Pollen and Vegetative Secondary Chemistry of Three Pollen-rewarding Lupines. *Am. J. Bot.* **2019**, *106*, 643–655, doi:10.1002/ajb2.1283.
27. Ne'eman, G.; Nesher, R. Pollination ecology and the significance of floral color change in *Lupinus pilosos L.* (Fabaceae). *Isr. J. Plant Sci.* **1995**, *43*, 135–145, doi:10.1080/07929978.1995.10676599.
